# Update on the Neurobiology of Vascular Cognitive Impairment: From Lab to Clinic

**DOI:** 10.3390/ijms21082977

**Published:** 2020-04-23

**Authors:** Luisa Vinciguerra, Giuseppe Lanza, Valentina Puglisi, Francesco Fisicaro, Manuela Pennisi, Rita Bella, Mariagiovanna Cantone

**Affiliations:** 1 Department of Neurology and Stroke Unit, ASST Cremona, 26100 Cremona, Italy; luisa.vinciguerra@asst-cremona.it (L.V.); valentina.puglisi@asst-cremona.it (V.P.); 2 Department of Surgery and Medical-Surgical Specialties, University of Catania, 95123 Catania, Italy; 3 Department of Neurology IC, Oasi Research Institute – IRCCS, 94018 Troina, Italy; 4 Department of Biomedical and Biotechnological Sciences, University of Catania, 95123 Catania, Italy; drfrancescofisicaro@gmail.com (F.F.); manuela.pennisi@unict.it (M.P.); 5 Department of Medical and Surgical Sciences and Advanced Technologies, University of Catania, 95123 Catania, Italy; rbella@unict.it; 6 Department of Neurology, Sant’Elia Hospital, ASP Caltanissetta, 93100 Caltanissetta, Italy; m.cantone@asp.cl.it

**Keywords:** small vessel disease, vascular dementia, cognition, plasticity, biochemistry, imaging, neuropathology, neurophysiology, treatment

## Abstract

In the last years, there has been a significant growth in the literature exploring the pathophysiology of vascular cognitive impairment (VCI). As an “umbrella term” encompassing any degree of vascular-related cognitive decline, VCI is deemed to be the most common cognitive disorder in the elderly, with a significant impact on social and healthcare expenses. Interestingly, some of the molecular, biochemical, and electrophysiological abnormalities detected in VCI seem to correlate with disease process and progression, eventually promoting an adaptive plasticity in some patients and a maladaptive, dysfunctional response in others. However, the exact relationships between vascular lesion, cognition, and neuroplasticity are not completely understood. Recent findings point out also the possibility to identify a panel of markers able to predict cognitive deterioration in the so-called “brain at risk” for vascular or mixed dementia. This will be of pivotal importance when designing trials of disease-modifying drugs or non-pharmacological approaches, including non-invasive neuromodulatory techniques. Taken together, these advances could make VCI a potentially preventable cause of both vascular and degenerative dementia in late life. This review provides a timely update on the recent serological, cerebrospinal fluid, histopathological, imaging, and neurophysiological studies on this “cutting-edge” topic, including the limitations, future perspectives and translational implications in the diagnosis and management of VCI patients.

## 1. Introduction

Vascular cognitive impairment (VCI) is considered as an “umbrella term” including a wide spectrum of cognitive disorders due to cerebrovascular disease of different etiologies, including large or strategic stroke, subcortical ischemic vascular disease (SIVD), multi-infarct strokes, border-zone ischemia, and intracerebral hemorrhages (especially large bleeding and multiple cortical and/or subcortical microbleeds) [1,2]. As such, VCI encompasses any degree of cognitive decline, ranging from the impairment of a single cognitive domain (mild VCI) to an overt vascular dementia (VaD) or mixed dementia (vascular and degenerative, most commonly of Alzheimer’s type) [3]. VaD represents the second most common cause of cognitive decline after Alzheimer’s disease (AD) and, among VaD subtypes, post-stroke dementia affects 15%-30% of patients 3 months after a stroke [4,5,6]. 

Several mechanisms link vascular disease to cognitive impairment. Historically, VaD has been associated with large vessel infarcts, however small vessel disease can also lead to cognitive decline and dementia [7]. A previous prospective study showed that at least seven different pathologies can predict VCI: large infarcts, lacunar infarcts, microinfarcts, myelin loss, arteriolosclerosis, cerebral amyloid angiopathy (CAA), and perivascular space dilation [8]. Among these, while large ischemic strokes or hemorrhages usually produce overt cognitive deficits due to gross lesions that overcome microstructural adaptations, in conditions of chronic microvascular damage, such as SIVD, leukoaraiosis, or microbleeds, molecular and synaptic alterations can contribute to a slowly progressive cognitive decline.

To date, the exact neurochemical basis underlying VCI is not completely clarified, although increasing evidence suggests that different components, i.e., oxidative stress, neuroinflammation, endothelial dysfunction, neurotransmitter imbalance, and cortical hyperexcitability play a role [1,2]. A better understanding of these mechanisms would be helpful for a more accurate identification and differential diagnosis of these patients, as well as for the development of evidence-based drugs and customized non-pharmacological treatments targeting specific markers of disease. 

In the last years, there has been a significant growth in the literature exploring VCI. In particular, some of abnormalities detected seem to correlate with the disease process and progression, eventually promoting adaptive plasticity in some patients and maladaptive, dysfunctional responses in others. However, the exact relationship between vascular lesion, cognition, and plasticity is not conclusively understood yet. Additionally, the possibility to identify specific subpopulations of subjects at risk for future dementia, although promising, still requires further clarifications. 

In the present review, we provided a multidimensional update on the recent studies on the biochemical basis of VCI, including the current limitations, future perspectives, and the translational implications in the diagnosis and management of these patients.

## 2. Data Source and Selection

A Medline (PubMed) literature review was performed by using the following search queries:

- ((“biochemistries”[All Fields] OR “biochemistry”[MeSH Terms]) OR “biochemistry”[All Fields]) AND ((“dementia, vascular”[MeSH Terms] OR (“dementia”[All Fields] AND “vascular”[All Fields])) OR “vascular dementia”[All Fields]);
- ((“biochemistries”[All Fields] OR “biochemistry”[MeSH Terms]) OR “biochemistry”[All Fields]) AND “vascular”[All Fields] AND ((((“cognitive dysfunction”[MeSH Terms] OR (“cognitive”[All Fields] AND “dysfunction”[All Fields])) OR “cognitive dysfunction”[All Fields]) OR (“cognitive”[All Fields] AND “impairment”[All Fields])) OR “cognitive impairment”[All Fields]);
- “cortical excitability”[MeSH Terms] OR (“cortical”[All Fields] AND “excitability”[All Fields]) OR “cortical excitability”[All Fields] AND (“dementia, vascular”[MeSH Terms] OR (“dementia”[All Fields] AND “vascular”[All Fields]) OR “vascular dementia”[All Fields]);
- “transcranial magnetic stimulation”[MeSH Terms] OR “transcranial magnetic stimulation”[All Fields] AND “vascular”[All Fields] AND ((((“cognitive dysfunction”[MeSH Terms] OR (“cognitive”[All Fields] AND “dysfunction”[All Fields])) OR “cognitive dysfunction”[All Fields]) OR (“cognitive”[All Fields] AND “impairment”[All Fields])) OR “cognitive impairment”[All Fields]).

Two independent authors (L.V. and V.P.) screened all titles and abstracts of the retrieved publications; disagreements were solved by the consensus of a third author (M.C.). Duplicated entries, retracted publications, studies on other diseases or conditions different from VCI or its subtypes, works on animals or in vitro, studies without statistical analysis, non-English written papers, publications that are not research studies (i.e., commentaries, letters, editorials, reviews, meta-analysis), and any other article that did not fit within the scope of this review, were excluded. Articles listed in the references were also reviewed in search of more data. Since we aimed to provide an update of the recent literature on the neurobiology of VCI by using modern investigation techniques, we considered only the studies published in the last decade (from January 2010 to March 2020).

## 3. Results

**Figure 1:**
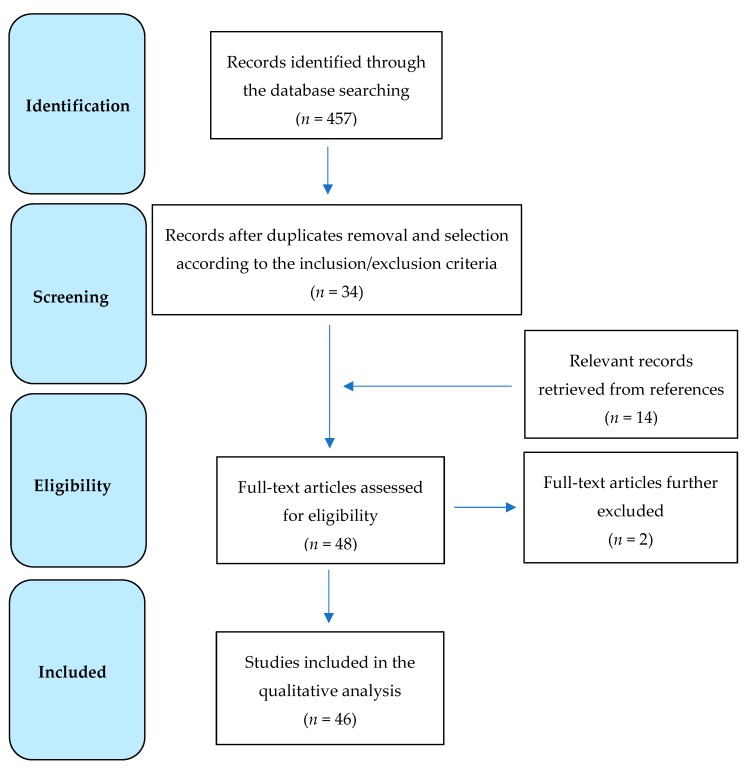
Flow diagram showing the search strategy, the number of records identified, and the number of included/excluded studies [55].

A total of 457 results were originally retrieved. Of these, 34 peer-reviewed publications were selected according to the above-mentioned inclusion and exclusion criteria. The examination of the references from relevant papers detected other 14 studies, whose analysis identified 12 additional articles fitting the purpose of this review. Therefore, a total of 46 papers were eventually included in this study (Figure 1) and the main findings are summarized in Table 1. More in details, 8 studies deal with serum biomarkers [9,10,11,12,13,14,15,16], 10 with cerebrospinal fluid (CSF) [17,18,19,20,21,22,23,24,25,26], 4 with neuroimaging [27,28,29,30], 6 with histopathology [31,32,33,34,35,36], and 14 with transcranial magnetic stimulation (TMS) [37,38,39,40,41,42,43,44,45,46,47,48,49,50]; 4 studies included more than one technique: one studied both serum and CSF markers [51], two both serum and neuroimaging [52,53], and one both CSF and neuroimaging [54].

### 3.1. Serum

Serological investigations represent a minimally invasive method to search biomarkers possibly identifying different types of dementia. Measures of oxidative stress, endothelial dysfunction, and inflammation, all deemed to play a role in VCI, can be feasibly evaluated by blood sampling. Changes in serum concentration of specific proteins might potentially act as markers of cognitive dysfunction or predict cognitive deterioration. The identification of any blood marker will implement the use of serological tests in clinical practice to early identify patients and possibly follow their progression.

Fleszar and colleagues demonstrated nitric oxide (NO)-related metabolite changes in VaD. In particular, citrulline, and dimethylarginine (DMA) concentrations were significantly higher in patients with strategic infarcts. Additionally, arginine depletion was an independent predictor of VaD, whereas DMA, asymmetric DMA (ADMA), and arginine/ADMA ratio were significant independent predictors of the variability of the Mini Mental State Examination (MMSE) score and Hachinski Ischemic Scale score [53]. S100B (calcium-binding protein B) is a protein that stimulates the expression of pro-inflammatory cytokines. Some authors demonstrated a significant correlation between S100B/ADMA levels and cognitive decline in patients with leukoaraiosis [11].

The anti-inflammatory and antioxidant functions of high-density lipoprotein, assessed through the dosage of the catalyzing enzyme, showed that low serum arylesterase levels were significantly associated with a higher probability of VaD [14]. Significant low arylesterase and paraoxonase activities were also observed in patients with mild cognitive impairment (MCI) who developed VaD, also predicting the risk of conversion after two years [13]. Additionally, simultaneously high levels of homocysteine and uric acid were associated with higher probability to develop VaD [9].

Homocysteine level, a well-known potential risk factor for vascular disease, resulted to be significantly associated with lacunar infarcts and deep cerebral microbleeds. Interestingly, the highest level significantly correlated with all causes of dementia, and especially of VaD, even after adjusting for age, gender, apolipoprotein E4 (APOE4), education, body mass index, MMSE, hypertension, cerebrovascular events, and estimated glomerular filtration rate [52]. These results were confirmed by Moretti and colleagues, who found a higher homocysteine level in VaD patients, but also a significantly vitamin D deficiency and low levels of folate [16], as well as in elderly patients with fatigue [56]. Liu and co-workers suggested a biomarker panel of three metabolites (glutamine, kynurenine, and lysophosphatidylcholines) to predict the post-stroke cognitive impairment [12].

Recently, a link between dementia and autoimmune disease has been hypothesized. Some authors detected that anti-smooth muscle antibodies were significantly associated with brain atrophy in different types of cognitive impairment, including VCI [15]. Increased levels of three proinflammatory cytokines (interleukin (IL)-1b, tumor necrosis factor-alpha (TNF-α), and interferon-gamma (IFN-γ)), two anti-inflammatory cytokines (IL-4 and IL-5), and one chemokine (granulocyte-colony stimulating factor (G-CSF)) were found in serum of VaD patients compared with AD and controls. Furthermore, cytokine levels of macrophage inflammatory protein-1beta (MIP-1β) and IL-8 correlated with neuropsychological test scores [51].

As markers of neuroinflammation and immune surveillance within the central nervous system (CNS), microglial cells resulted to be activated in MCI patients evolving in VaD [23]. These patients also demonstrated abnormalities of proteins involved in extracellular matrix synthesis/degradation, angiogenesis, axonal damage, hemostatic processes, and inflammatory responses [1,17,22].

Finally, in a large population prospective study over a median of 13 years, subclinical myocardial injury, assessed by higher baseline concentration of the high-sensitivity cardiac troponin T, was significantly associated with increased risk of VaD and hospitalization due to dementia [10].

### 3.2. Cerebrospinal Fluid

CSF analysis is a very useful exam for the early diagnosis and prognosis of a wide range of neurological conditions, including cerebrovascular diseases and neurodegenerative disorders [57,58]. CSF abnormalities, indeed, reliably reflect pathological changes within the CNS and, interestingly, these changes might be detected even years before the clinical onset of a cognitive disorder [55,56]. For these reasons, CSF analysis can help in disentangling the complex mechanisms underlying dementia and in distinguishing among different forms of cognitive impairment.

CSF total tau (T-tau), phosphorylated tau (P-tau), and amyloid-beta (Aβ) peptides have been proposed as discriminating measures between AD and non-AD dementias, including VCI. Nevertheless, the heterogeneity of VCI construct often makes hard this distinction, as well as to distinguish between different forms of VaD based on CSF markers alone. However, CSF analysis may increase the diagnostic accuracy of VaD based on the evidence that some measures (e.g., CSF total protein) and indexes (e.g., albumin ratio) have been associated with both structural and functional changes of the blood–brain barrier (BBB) and microvascular damage [59]. In this framework, as markers for axonal damage in response to small vessel disease, the increase of neurofilament-light protein (NF-L) levels was significantly correlated with vascular white matter lesions (WMLs) in patients with cognitive decline. Instead, T-tau, P-tau, Aβ markers, and CSF/serum albumin ratio were not associated to WMLs severity in VCI [17]. 

In a pure genetic model of vascular dementia, i.e., the cerebral autosomal dominant arteriopathy with subcortical infarcts and leukoencephalopathy (CADASIL), T-tau and P-tau levels were normal, whereas Aβ42 levels were significantly lower with respect to controls [18]. These findings were confirmed by Spies and co-workers who found the Aβ42 and Aβ42/Aβ40 ratio higher in VaD compared to AD patients [19], although in another study decreased Aβ42 and increased tau were similarly found both in VaD and AD [24]. Nevertheless, Aβ42, Aβ40, P-tau, and Aβ42/Aβ40 ratio were similar between dementia due to CAA and AD [25], thus suggesting that this ratio could not differentiate between these two forms of dementia. 

Notably, a positive correlation between Aβ40, T-tau, P-tau, and CSF-cellular prion protein levels was observed in VaD patients [51]. Additionally, the increase of selected protein panel, such as Aβ1-42, T-tau, P-tau, myelin basic protein, tissue inhibitors of metalloproteinases-1 (TIMP-1), NF-L, matrix metalloproteinases (MMP)-9, and MMP-2, was found to be helpful in differentiating SIVD patients with cognitive decline from AD, with a sensitivity of 89%, a specificity of 90% [20]. Furthermore, VCI subjects exhibited a significant increased MMP-3 activity and a reduced MMP-2 index (measured by the MMP-2 levels in blood and CSF) compared to controls, and the combination of these two parameters showed a high specificity to distinguish SIVD patients from controls [21]. Reduced MMP-2 index and elevated albumin index were also able to reliably predict the development of Binswanger disease in VCI [26]. Similarly, albumin index resulted significantly increased in SIVD [54].

Finally, some authors found significantly elevated microglial markers, such as YKL-40 (also known as chitinase-3-like protein 1) and soluble cluster of differentiation 14 in MCI subjects who developed VaD [23]. Other CSF proteins, like alpha1-antitrypsin (AAT), apolipoprotein H (APOH), plasminogen activator inhibitor-1 (PAI-1), and heart-type fatty acid binding protein (H-FABP), resulted increased in MCI that converted into subcortical ischemic VaD [22].

### 3.3. Neuroimaging 

Imaging is crucial in the diagnosis and management of dementia, particularly in VCI patients, due to the clear visualization of vascular injury of both grey and white matter and cortical-subcortical atrophy. Some authors evaluated the cholinergic pathway involvement in VCI by assessing the hyperintense signal change at magnetic resonance imaging (MRI) thorough a standardized visual rating scale (the Cholinergic Pathway HyperIntensity Scale). Of note, they found that the ischemic damage within cholinergic circuits was correlated with dementia severity in SIVD patients and, in particular, in those with frontal lobe cognitive impairment [29]. Conversely, L-arginine/NO pathway metabolites resulted to be not different with respect to Fazekas score [53].

Brain metabolites detected with magnetic resonance spectroscopy (MRS) demonstrated to correlate with neuropsychological test scores. Namely, total creatine and *N*-acetyl-aspartyl compounds in cerebral white matter were significantly associated with overall T score and executive function, even after adjusting for lesion volume [30]. 

Brain glucose metabolism evaluated by 18-fluoro-deoxyglucose (FDG)-positron emission tomography (PET) showed different abnormalities in VCI compared to AD. Namely, FDG distribution in vascular WMLs patients with dementia was altered in the caudate nucleus, thalamus, and frontal lobe [27]. More recently, the Pittsburgh compound B (PIB)-PET has emerged as an advanced imaging technique able to detect amyloid plaques and accumulation in different types of dementia. PIB-PET demonstrated to be positive in post-stroke dementia and the accumulation might be associated with a more rapid cognitive decline [28]. This suggests that amyloid pathology and vascular lesion may coexist and interact each other by increasing the severity of cognitive manifestations, as typically observed in patients with mixed dementia.

Dysfunction of the neurovascular unit may contribute to the amyloid accumulation in addition to a direct neuronal damage, i.e., through the disruption of the BBB and a decrease in cerebral blood flow (CBF) [60,61,62]. Further, changes of the brain glucose metabolism were particularly evident in the frontal lobe of VaD patients, thus providing a metabolic pattern of cognitive impairment different from that observed in AD [27]. In addition, regional distribution differences of some MRS-related metabolites resulted associated with executive dysfunction, which is commonly present in VCI patients, thus being useful in distinguishing different types of dementia also at the neuropsychological level [30]. Finally, it has also been shown that amyloid plaque retention was identified in post-stroke patients who developed a more rapid cognitive decline [28].

Regions of neuroinflammatory disruption of BBB, quantified and visualized by dynamic contrast-enhanced MRI [63], resulted in a significantly increased BBB permeability in VCI patients compared to controls [54]. Moreover, cerebral hypoperfusion as a consequence of microangiopathy, macroangiopathy or cardiac failure, can promote or accelerate BBB disruption, neuroinflammation, and neurodegeneration [64,65]. In this context, the arterial spin labelling (ASL) is a modern MRI technique that allows brain perfusion to be measured non-invasively at the tissue level and to assess intracranial vascular compliance [66]. A widespread decrease in CBF has been found in VaD, especially in bilateral frontal and parietal areas, but also in confluent WMLs [67] and post-stroke patients [68]. Moreover, ASL patterns seem to follow the progression of cognitive decline, suggesting a promising role as a biomarker of disease progression [64]. However, due to the low perfusion levels and long arterial arrival times in the white matter, to date the ASL is considered not as sensitive to perfusion changes in the deep white matter as in the cortex [69].

### 3.4. Transcranial Magnetic Stimulation 

Among neurophysiological techniques, TMS is a non-invasive tool evaluating cortical excitability, synaptic plasticity, and functional connectivity in vivo and in “real time”, in physiological conditions [70,71,72] and in a number of psychiatric and neurological disorders [73,74,75,76,77,78], including cognitive impairment and dementia [42,49,79], also providing neurochemical insights [80,81,82,83].

The majority of TMS studies in VaD indicate that the motor cortex is hyperexcitable in terms of reduced resting motor threshold (rMT) [84,85], which is a basic feature also shared by AD [86], suggesting a compensatory mechanism in response to ischemic injury and neuronal loss [87,88]. Translationally, this finding has been viewed as the correlate of impaired excitatory glutamatergic transmission [89,90], although a reduced inhibitory effect cannot be excluded, which, in turn, would facilitate the cortico-spinal output [87]. On the other hand, gamma-amino-butyric acid (GABA) release in response to glutamate overactivation is considered part of the mechanisms of neuronal defense to compensate for excitotoxicity [91]. 

Conversely, the short-latency afferent inhibition (SAI), which is an index of central cholinergic transmission [70], was found significantly reduced in the subcortical ischemic VaD, although individual data varied widely [92]. In a different study, indeed, mean SAI was reduced in AD and normal in VaD, except for 25% of VaD patients who probably had a mixed dementia [93]. In a more recent paper in mild VCI with predominant WMLs, SAI was not clearly affected [49], thus suggesting a distinctive profile with respect to AD and MCI [94]. A reasonable explanation of this result is that VCI patients often exhibit great interindividual variations in the location and severity of subcortical infarcts and, therefore, in the distribution and magnitude of the cholinergic denervation [95].

In this scenario, a crucial issue is whether it is possible to identify mild VCI subjects at risk for VaD. In a previous study in this population [40], it was found that the ischemic interruption of prefrontal-subcortical loops implicated in executive functions and mood/affection regulation resulted in an enhanced glutamate-mediated index of intracortical facilitation (ICF) [40]. A two-year follow-up study in the same patients showed an increase of the global cortical excitability (reduction of the median rMT), along with a significant cognitive worsening [41], thus suggesting the role of the enhanced ICF at baseline as a predictor of cognitive and TMS progression. Finally, unlike MCI and AD, TMS measures of transcallosal functioning appeared to be spared in mild VCI, suggesting a role of the interhemispheric integrity in holding cognitive performance up in these patients [42].

Other supportive data come from the repetitive TMS (rTMS), which is a paradigm of stimulation that non-invasively and transiently modulates cortical circuits and related neurochemical pathways [96,97,98], even with long-lasting effect [99,100]. In a randomized controlled pilot study, Rektorova and colleagues [101] showed that high-frequency (excitatory) stimulation over the left dorsolateral prefrontal cortex improved executive performance and hypothesized that long-lasting effects might be due to an indirect activation of monoaminergic neurons located in the midbrain (dopamine) and/or brainstem (noradrenaline and serotonin) and their cortical and subcortical targets [101].

The few TMS studies in CADASIL converge on a profile of cortical hyperexcitability, probably due to a glutamatergic and GABAergic dysfunction, as observed in other dementias [79,80,102,103]. More recently, SAI was found significantly reduced both in AD and CADASIL, although the administration of levo-3,4-dihydroxyphenylalanine (L-DOPA) increased it in the AD group only, thus hypothesizing that the relationship between acetylcholine and dopamine systems may be specifically abnormal in AD only [46]. The study of intracortical circuits to paired-pulse TMS and sensory-motor plasticity to paired associative stimulation (PAS) confirmed that acetylcholine and glutamate are involved and that abnormal plasticity correlates with neuropsychological scores [44].

### 3.5. Histopathology 

Histological assays, although invasive and therefore usually performed postmortem, allow a direct examination of neuronal and glial cells, as well as of the level and structure of different extracellular constituents, thus providing an objective assessment of the type, location, and extent of vascular lesions and cortical–subcortical atrophy. Moreover, enzymatic activity, synaptic morphology, and some mechanisms underlying neurovascular dysfunction (e.g., cellular permeability, regeneration, adhesion molecules, and endothelial components) can be displayed, thus providing both quantitative and qualitative information on vascular and parenchymal changes.

The vesicular glutamate transporter 1 (VGLUT1) has been related to synapses crucially involved in cognitive processes [31]. In particular, VGLUT1 concentration in the Brodmann area 9 of neocortical region significantly differed between stroke patients who did not develop dementia and those with dementia of different types (VaD, AD, and mixed VaD/AD), even after the Bonferroni correction [31]. Furthermore, the concentration of synaptophysin, which is a neuronal synaptic vesicle that participates in synaptic transmission [104], was higher in the temporal cortex of VaD group with respect to AD, as well as in stroke subjects who did not develop dementia [31]. Conversely, Sinclair and collaborators found significantly lower synaptophysin with lower synaptosomal-associated protein 25 and enhanced drebrin levels in the superior temporal cortex of VaD patients than controls [35]. 

Other relevant pathological substrates of VCI patients were observed in the frontal lobe, including vacuolation, dendritic process fragmentation, cytoplasmic swelling of the astrocyte soma [36], and reduced myelin density [32]. These findings demonstrated the highly increased degeneration of astrocytes and oligodendrocytes shrink due to ischemia and axonal degeneration. The examination of brain coronal sections showed that clasmatodendrocyte percentage in the deep frontal white matter regions, particularly at the level of the middle frontal gyrus, the anterior horn of the lateral ventricle, and the centrum semiovale, was significantly greater in the post-stroke dementia subjects than in the post-stroke group without dementia. Clasmatodendritic cells were also swollen and had a vacuolated appearance [36]. Additionally, the frontal but not the temporal lobe exhibited significant myelin attenuation with reduced myelin density in VaD compared to controls, AD, and Lewy body dementia (LBD). Finally, in VaD cases, the mean percentage area containing the degraded myelin basic protein was inversely correlated with the mean size of oligodendrocytes [32]. 

As indicator of pathological cortex hypoperfusion, the myelin associated glycoprotein to proteolipid protein 1 ratio was significantly reduced in VaD patients compared to controls [34]. On the other hand, substantial neuron loss in the nucleus basalis of Meynert or chromatolysis as sign of retrograde degeneration were not observed in SIVD patients [33].

## 4. Discussion

### 4.1. Summary of Findings

From the studies reviewed here, it appears evident that several regulatory mechanisms and cellular signaling, including, oxidative stress, neuroinflammation, endothelial dysfunction, hypoperfusion, BBB disruption, cortical hyperexcitability, and neurotransmitter imbalance are all deemed to play a role in VCI. Although some of these pathomechanisms are also shared by AD, the findings seem to converge on the possibility that some changes might be specifically involved in VCI patients. Overall, the level of specific proteins in CSF and the findings coming from advanced neuroradiological techniques and histopathology assays are able to reveal some diagnostic features of VCI. However, blood-based and TMS results, although currently lacking of the same sensitivity, specificity, and positive predictive value, represent the more minimally invasive and cost-effective methods to potentially identify and follow-up VCI in clinical practice. 

Some studies have identified serological markers that can support the diagnosis of VaD. For instance, pro-inflammatory metabolites (such as NO-related molecules), cytokines (including IL-1β, TNF-α, IFN-γ, IL-4, IL-5, IL-8, G-CSF, and MIP-1b), and markers of endothelial dysfunction (e.g., homocysteine) were found to be increased in VaD patients [11,16,51,52,53]. Furthermore, an altered systemic redox balance was demonstrated in VCI, in which reduced antioxidant enzymes, such as the activity of arylesterase and paraoxonase, were associated with the risk of developing dementia [13,14]. In addition, the concentrations of some CSF proteins can also help to distinguish among different forms of dementia. In particular, the analysis of a specific protein panel, including Aβ42/Aβ40 ratio, Aβ42, P-tau, T-tau, TIMP-1, NF-L, MMP-9, and MMP-2, was able to reliably distinguish VaD from either AD, LBD, or normal aging [19,20,21,105]. Further, a disruption of BBB integrity was demonstrated in VCI patients through the increase of the CSF/serum albumin ratio [54]. Notably, a BBB breakdown has been demonstrated also in AD [106], although, VCI patients had higher CSF albumin levels compared to AD. Moreover, among VCI subtypes, SIVD had the highest value of mean BBB permeability and albumin index, thus suggesting a linking role of BBB dysfunction between vascular lesion and tissue damage [54,107,108].

Histological data confirmed most of the findings that have been observed or hypothesized in vivo. In particular, the glutamatergic synapse loss, especially found in the frontal cortex, is related to cognitive dysfunction in both VaD and AD, whereas the preservation of glutamatergic synapses plays a role in supporting cognition and in protecting against dementia after a stroke. Indeed, the VGLUT1 up-regulation seems to correlate with preserved cognitive function in subjects with cerebrovascular disease [31,109]. As known, the disruption by WMLs or lacunar infarcts of the frontal subcortical circuits implicated in cognition and mood-affect regulation may primarily result in executive dysfunction, attention deficit, apathy, and drug-resistant late-onset depression, that are commonly observed in VCI with SIVD [110,111]. 

Notably, although mitochondrial dysfunction is a well-established hallmark of Aβ-induced neuronal toxicity [112], several mitochondrial mechanisms are invoked also in Aβ-related cerebrovascular degeneration. Accordingly, mitochondrial DNA mutations may have a cumulative effect by increasing the probability to develop an energy failure and possibly lowering the age of onset of VaD [113], as already described in degenerative dementia [114]. 

Neuropathological data also supports the neurophysiopathology of these patients. Indeed, TMS points at enhanced cortical excitability in VaD, which may suggest a glutamate-mediated compensatory effects in response to vascular lesions [115]. Based on these results, it has been hypothesized the enhanced excitability and subsequent plasticity might counteract cognitive decline [39,41]. This has been clearly demonstrated by TMS mapping studies in AD patients, who showed a frontal and medial shift of the center of gravity of the motor cortical representation map, suggesting a functional reorganization of specific cortical brain areas [116,117]. More recently, a similar pattern has been observed also in subcortical ischemic VaD [37,48], a subtype of VCI with insidious onset, gradual course, and slow progression, often making challenging the differentiation from AD [5,118].

Interestingly, TMS findings point out the possibility to predict the progression of mild VCI into VaD, especially in those with concomitant late-onset depression and SIVD (the so-called “vascular depression”) [39,50], and to select responders to specific drugs or non-pharmacological treatments [40,41,49,85]. This will be of pivotal importance when designing trials of disease-modifying drugs or approaches based on rTMS or other neuromodulatory interventions [119]. 

Recently, it has been proposed that a mechanism of action of non-invasive brain stimulation techniques in dementia may be the modulation of neurotrophin release, although systematic studies in humans are still lacking. In a murine model of VaD [120,121], low-frequency (inhibitory) rTMS was able to improve cognitive deficit through the up-regulation of the hippocampal brain-derived neurotrophic factor (BDNF) and the expression of the *N*-methyl-d-aspartate glutamate receptor [122]. Moreover, low-frequency rTMS in VaD rats improved learning and memory, protected pyramidal cells from apoptosis, and promoted hippocampal synaptic plasticity through the increased expression of the *Bcl-2* (B-cell lymphoma 2) gene and reduced expression of the *Bax* (BCL2 associated X protein) gene [123]. A mechanism likely related to the promotion of BDNF expression and subsequent restoration of cholinergic system activity in hippocampal CA1 region was also observed [124]. Finally, a synergistic effects of mesenchymal stem cell transplantation and rTMS on promoting autophagy and synaptic plasticity in VaD rats has been demonstrated [125]. 

At the level of structural and functional neuroimaging in VCI, an impairment of cholinergic networks was detected in these patients and was particularly related to frontal cognitive dysfunction [29]. It is known that penetrating arteries supplying cholinergic basal forebrain nuclei are particularly vulnerable to the arterial hypertension because of their anatomical distribution arising directly from carotid system [105,126]. Additionally, since cholinergic pathways are involved in the regulation of CBF [127,128], cholinergic-based abnormalities might potentially lead to hypoperfusion and contribute to the pathogenesis of VaD [2,129]. 

Finally, previous studies have applied transcranial Doppler ultrasound (TCD) to explore the relationship between cerebral hemodynamics and brain lesions attributed to small vessel disease in cognitive disorders [64,130,131,132]. As known, TCD is a non-invasive and feasible neurosonological technique able to evaluate CBF velocity, arterial perfusion integrity, and intracranial small vessel compliance [133,134]. The microangiopathy, demonstrated both in VaD and AD, might lead to arteriolosclerosis, vasoconstriction, and vascular stiffness, thus resulting in decreased arterial diameter and CBF [64,135,136]. In a recent TCD study [137], mild VCI patients showed a hemodynamic pattern of cerebral hypoperfusion and enhanced vascular resistance, likely arising from small vessels and then extending to larger arteries. This result provides evidence of the occurrence and severity of small vessel disease and executive dysfunction in elderly patients at risk of future dementia [137]. It has been also demonstrated that a similar hemodynamic dysfunction might play a pathogenic role in the development of cognitive impairment in patients with vascular depression and predominant WMLs [138]. Further studies aiming at a direct TCD comparison between AD and VaD, and their preclinical stages (i.e., MCI and VCI, respectively), are warranted.

### 4.2. Limitations and Future Directions

Although comprehensive, the approach used in the examined investigations in the attempt to disentangle the complex pathomechanisms of VCI has a number of caveats and potential criticisms.

First, is the heterogeneous construct of VCI, which still constitutes a challenge for clinicians and researchers in the patients’ selection and identification of appropriate outcome measures, also in trials of pharmacological interventions. In this context, patient cohorts and methodologies are not always homogeneous across studies, and a single diagnostic method is not sufficient to define a diagnosis.

Second, the difficulty in recruitment of a sufficient number of age-matched controls without evidence of cerebrovascular disease at neuroimaging (that is strikingly prevalent among elderly) or cognitive impairment at the neuropsychological evaluation. Therefore, the available results on relatively small sample size might not be confirmed on larger populations, although most of them were obtained from homogeneous samples in terms of demographics, clinical, and neuroradiological features, and were age-matched with healthy controls.

Another limitation is that the correlation between different techniques and the anatomical distribution and severity of vascular lesions has been rarely systematically investigated; therefore, without the contribution of advanced imaging, neuronavigational systems, or the combination of techniques, the conclusions that can be reached cannot be sufficiently powerful. 

Fourth, results do not usually provide specific clinical information, although they are sensitive to the “global weight” of several biochemical pathways and neurotransmitter activities, as well as to subcortical and cortical inputs. As a consequence, the identification of a clinical correlate of VCI is often challenging and, as a general rule, cannot be linked to a specific VCI subtype. In this scenario, the hypothesis to identify a characteristic signature in patients with cerebrovascular disease at risk for VaD or mixed dementia might be risky given the paucity of previous data and the difficulty of similar approaches in other cognitive disorders, such as non-AD dementia or secondary dementia. Consequently, a panel of changes, rather than single marker of disease, should be considered.

Fifth, it is known that vascular lesions, even in the absence of any motor deficit, give a significant contribution to the development and progression of degenerative dementia (most commonly of AD), so that it cannot be excluded that at least some patients enrolled in the studies here reviewed actually had a mixed form of dementia rather than a pure VaD. In this respect, some of the techniques described (e.g., neuroimaging and TMS) are not currently able to clearly distinguish VaD from AD or to reliably disentangle the vascular from the degenerative burden. 

Another caveat is that elderly population usually takes a number of drugs for the treatment of vascular risk factors (e.g., antithrombosis agents, antihypertensive drugs, statins, and oral antidiabetic therapy) which may affect the reliability and reproducibility of the results. The same holds for psychotropic medications often taken for the treatment of comorbid anxiety and/or depressive disorders (e.g., benzodiazepines, antidepressants, antipsychotics, and mood stabilizers). Therefore, the study design should consider patients under medicaments which, to the best of the current knowledge, might have no or minimal influence on the technique considered (e.g., functional neuroimaging and TMS).

Finally, a usual limitation in this type of research is the threshold of sensitivity and specificity of the method employed. In order to adequately define sensitivity and specificity, the individual measures in all the patients and controls, and not only the mean values, would be required. Moreover, to estimate the number of false positives, the whole subject population needs to be independently followed up. Up to now, however, very few studies fulfil these requirements and the application of different diagnostic approaches in dementia need future studies with methodological improvement and high degree of standardization. 

**Figure 2:**
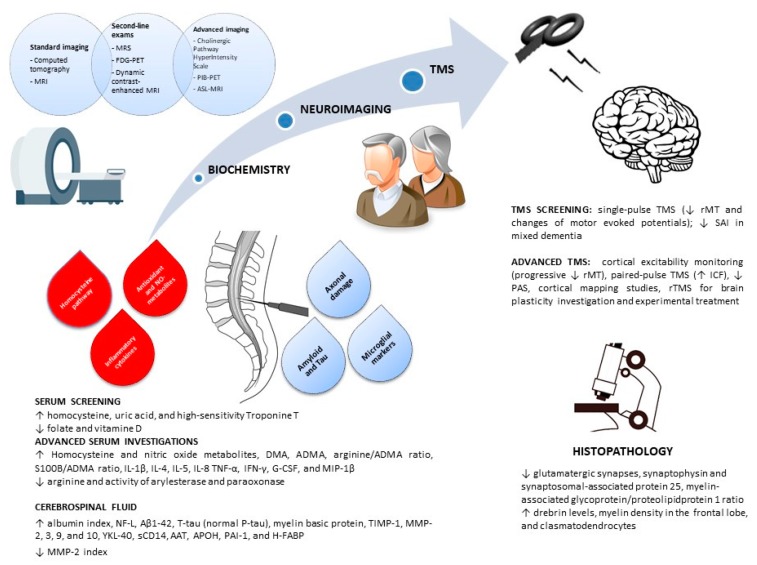
Proposed multidimensional diagnostic panel/algorithm for VCI subjects. Legend (in alphabetical order): Aβ: amyloid beta; AAT: alpha1-antitrypsin; ADMA: asymmetric dimethylarginine; APOH: apolipoprotein H; ASL: arterial spin labelling; DMA: dimethylarginine; FDG-PET: 18-fluorodeoxyglucose-positron emission tomography; G-CSF: granulocyte-colony stimulating factor; H-FABP: heart-type fatty acid binding protein; ICF: intracortical facilitation; IFN-γ: interferon gamma; IL: interleukin; MIP-1β: macrophage inflammatory protein 1beta; MMP: matrix metalloproteinases; MRI: magnetic resonance imaging; MRS: magnetic resonance spectroscopy; NF-L: neurofilament-light protein; P-tau: phosphorylated tau; PAI-1: plasminogen activator inhibitor 1; PAS: paired-associative stimulation; rMT: resting motor threshold; rTMS: repetitive transcranial magnetic stimulation; S100B: calcium-binding protein B; SAI: short-interval afferent inhibition; sCD14: soluble cluster of differentiation 14; T-tau: total tau; TIMP-1: tissue inhibitors of metalloproteinases 1; TMS: transcranial magnetic stimulation; TNF-α: tumor necrosis factor alpha; YKL40: chitinase-3-like protein 1.

For all the above-mentioned reasons, the role of these measures as possible biomarkers of VCI, as well as the possibility that they might represent an epiphenomenon of vascular damage rather than own a diagnostic value, is still under debate. This review might consolidate, wherever possible, the available knowledge and stimulate further research. In Figure 2, a diagnostic panel/algorithm for VCI subjects is proposed, whereas Table 2 summarizes the research agenda and future avenues of research that might be developed for a further translation of VCI biomarkers into clinical practice.

## 5. Conclusions

To date, the development of dementia cannot be accurately predicted by conventional investigations. However, unlike degenerative dementia, VaD can be prevented, at least in part, in most of the patients through a careful prevention and a close monitoring of vascular risk factors. Preventing VCI means to prevent vascular accidents and future dementia, to reduce mortality, disability, and institutionalization, to maintain an acceptable functional status in the elderly, and, ultimately, to save healthcare costs and social expenses. 

The studies reviewed here suggest that, notwithstanding their limitations, all the findings of interest can be considered as footprints of the pathophysiological processes that affect VCI and, some of these, might possibly predict the conversion of mild VCI into VaD. This will be of pivotal importance when designing trials of disease-modifying drugs and innovative non-pharmacological approaches. In patients with overt dementia, these biomarkers can be exploited to select and evaluate the responders to specific drugs and rehabilitative strategies in the attempt to restore impaired plasticity. Future multidimensional studies and follow-up combining different techniques and outcome measures will shed further lights on this complex fascinating topic.

## Figures and Tables

**Table 1:** Recent multidimensional studies on the neurobiology of vascular cognitive impairment.

Authors, Year	Study Design	Patients (n).	VCI Subtype	Main Findings
***Serum***				
Cervellati, et al. 2014 [9]	Cross-sectional	54	Not reported	↑ homocysteine and uric acid ↓ residual antioxidant power ↑ slightly of hydroperoxide level
Schneider, et al. 2014 [10]	Prospective	34	Not reported	High-sensitivity cardiac troponin T associated with increased risk of vascular dementia
Gao, et al. 2015 [11]	Cross-sectional	210	Isolated or multiple lacunar infarcts; leukoaraiosis	↑ plasma levels of S100 Calcium-Binding protein B and asymmetric dimethylarginine associated with small vessel disease in patients with cognitive decline
Liu, et al. 2015 [12]	Cross-sectional	30	Not reported	↑ carnitine, glutamine, uric acid, tyrosine, kynurenine, and phenylalanine ↓ 3-indolepropionic acid, stearoyl-carnitine, valine, isoleucine, tryptophan, lysophosphatidylcholines, and palmitoylcarnitine
Cervellati, et al. 2015 [13]	Case-control	21	Not reported	↓ paraoxonase and arylesterase activity, which was associated with risk of conversion of mild cognitive impairment into vascular dementia
Castellazzi, et al. 2016 [14]	Cross-sectional	65	Not reported	↓ serum arylesterase level = paraoxonase level
Paroni, et al. 2016 [15]	Cross-sectional	31	Cortical/subcortical or strategic infarcts	Anti-smooth muscle antibody associated with brain atrophy
Moretti, et al. 2017 [16]	Cross-sectional	456	White matter lesions	↓ folate and vitamin D ↑ homocysteine
***Cerebrospinal fluid***				
Jonsson, et al. 2010 [17]	Cross-sectional	53	White matter lesions	↑ neurofilament light protein associated with white matter lesion severity; less strong evidence for sulfatide
Formichi, et al. 2010 [18]	Cross-sectional	10	CADASIL	↓ amyloid-beta 42, overlapping to Alzheimer’s disease = total tau and phosphorilated tau, which differed from Alzheimer’s disease
Spies, et al. 2010 [19]	Cross-sectional	26	Not reported	↑ amyloid-beta 42 and amyloid-beta 42/amyloid-beta 40 ratio in vascular dementia than Alzheimer’s disease
Bjerke, et al. 2011 [20]	Case-control	26	SIVD	↑ major basic protein, neurofilament light protein, heart-fatty acid binding protein, total tau, tissue inhibitor of metalloproteinases-1, and matrix metalloproteinase-10
Candelario-Jalil, et al. 2011 [21]	Cross-sectional	60	SIVD; multiple strokes; leukoaraiosis	↓ matrix metalloproteinase-2 index, with a negative correlation with albumin ratio ↑ matrix metalloproteinase-3 activity
Öhrfelt, et al. 2011 [22]	Cross-sectional	8	White matter lesions; lacunar infarcts	↑ alpha1-antitrypsin, apolipoprotein H, plasminogen activator inhibitor-1, heart-fatty acid binding protein, and tissue inhibitor of metalloproteinases-1
Olsson, et al. 2012 [23]	Prospective	19	SIVD	↑ chitinase-3-like protein 1 and soluble cluster of differentation 14; chitinase-3-like protein 1 differentiated between stable mild cognitive impairmennt and those converting into Alzheimer’s disease and vascular dementia ↑ cerebrospinal fluid/serum albumin ratio
Kaerst, et al. 2013 [24]	Retrospective case-control	44	Not reported	↓ amyloid beta 42
Li, et al. 2015 [25]	Cross-sectional	5	Cerebral amyloid angiopathy	= amyloid-beta 42, amyloid-beta 40, phosphorylated tau, and amyloid-beta 42/amyloid-beta 40 ratio between cerebral amyloid angiopathy and Alzheimer’s disease
Rosenberg, et al. 2015 [26]	Prospective	62	SIVD	↑ albumin index ↓ matrix metalloproteinase-2 index
***Neuroimaging***				
Pascual, et al. 2010 [27]	Cross-sectional	12	Confluent white matter lesions	↓ metabolism in both frontal lobes and right supramarginal gyrus at the 18-fluoro-deoxyglucose-positron emission tomography
Mok, et al. 2010 [28]	Cross-sectional	10	SIVD; large post-stroke lesions	Pittsburgh compound B-positron emission tomography binding was commonly observed
Kim, et al. 2012 [29]	Cross-sectional	48	White matter lesions	Cholinergic pathway deficit
Gasparovic, et al. 2013 [30]	Cross-sectional	60	Multiple stroke; SIVD; hypoxic hypoperfusion	Correlation between total creatine, N-acetyl-aspartyl compounds and test scores of executive function; metabolite levels correlated with total white matter lesion volume
***Combined serum and cerebrospinal fluid***				
Schmitz, et al. 2015 [51]	Cross-sectional	42	SIVD	Interleukin-8 and macrophage inflammatory protein-1β correlated with dementia severity; association between cellular prion protein and cytokine levels and between cellular prion protein and degenerative marker proteins
***Combined serum and neuroimaging***				
Miwa, et al. 2015 [52]	Case-control	18	SIVD	Total homocysteine level contributed to the increased risk susceptibility of dementia
Fleszar, et al. 2019 [53]	Cross-sectional	40	Strategic infarcts; white matter lesions	↑ dimethyarginine, L-citrulline, asymmetric dimethylarginine, and symmetric; dimethyarginine, L-arginine/ asymmetric dimethylarginine, and dimethyarginine independently predicted Hachinski Ischemic score
***Combined cerebrospinal fluid and neuroimaging***				
Taheri, et al. 2011 [54]	Cross-sectional	60	SIVD; multiple and lacunar infarcts; leukoaraiosis	↑ albumin index and blood–brain barrier permeability
***Transcranial magnetic stimulation***				
Pennisi et al. 2011 [37]	Cross-sectional	20 vascular dementia 20 mild VCI	SIVD dementia	↑ cortical excitability in demented patients only
Nardone, et al. 2011 [38]	Cross-sectional	28	SIVD dementia	Microbleeds on cholinergic function are independent of white matter lesion extent and ischemic stroke
Bella, et al. 2011 [39]	Cross-sectional	15 major depressive disorder 10 non-depressed	SIVD mild VCI	Neurophysiology of vascular depression differs from major depressive disorder and seems to be similar to that of SIVD
Bella, et al. 2011 [40]	Cross-sectional	10	SIVD mild VCI	↑ intracortical excitatory neuronal circuits
Bella, et al. 2013 [41]	Case-control	9	SIVD mild VCI	↑ excitability during the progression of VCI
Lanza, et al. 2013 [42]	Cross-sectional	15	Leukoaraiosis mild VCI	= transcallosal inhibitory functioning, unlike Alzheimer’s disease and mild cognitive impairment
List, et al. 2013 [43]	Cross-sectional	20	Leukoaraiosis mild VCI	↑ cortical plasticity as a compensatory mechanism
Palomar, et al. 2013 [44]	Cross-sectional	10	CADASIL	Acetylcholine and glutamate involved; abnormal sensory-motor plasticity correlated with cognition
Concerto, et al. 2013 [45]	Cross-sectional	11 depressed 11 major depressive disorder	SIVD mild VCI	Distinctive patterns of cortical excitability between late-onset vascular depression and early-onset non-vascular major depressive disorder
Nardone, et al. 2014 [46]	Cross-sectional	8 VCI 8 Alzheimer’s disease	CADASIL	↓ cholinergic functioning, with restoration by L-3,4-dihydroxyphenylalanine in Alzheimer’s disease only
List, et al. 2014 [47]	Cross-sectional	12	Mild VCI (post-stroke in 3 of them)	↓ long-term potentiation-like plasticity in the affected hemisphere = motor learning between hemispheres, maybe due to γ-amino-butyric acid B-effect in the affected side
Guerra, et al. 2015 [48]	Cross-sectional	7 VCI 9 Alzheimer’s disease	SIVD dementia	↑ excitability and plasticity in Alzheimer’s disease and vascular dementia; the hyperexcitability promoted plasticity
Bella, et al. 2016 [49]	Cross-sectional	25	SIVD mild VCI	Central cholinergic pathway not clearly affected
Pennisi, et al. 2016 [50]	Case-control	16 major depressive disorder 11 non-depressed	SIVD mild VCI	↑ risk of dementia in vascular depression, probably due to subcortical vascular lesions or to the lack of compensatory functional cortical changes
***Histopathology***				
Kirvell, et al. 2010 [31]	Case-control	18	Not reported	↓ glutamatergic synapses
Ihara, et al. 2010 [32]	Case-control	20	White matter lesions	↓ myelin density in the frontal lobe
Jung, et al. 2012 [33]	Case-control	16	SIVD	No neuron loss in the nucleus basalis of Meynert; no evidence of retrograde degeneration
Thomas, et al. 2015 [34]	Case-control	17	Not reported	↓ myelin-associated glycoprotein/proteolipidprotein 1 ratio
Sinclair, et al. 2015 [35]	Case-control	11	Multiple infarcts	↓ synaptophysin and synaptosomal-associated protein 25 ↑ drebrin levels
Chen, et al. 2016 [36]	Case-control	27	Multiple infarcts; lacunae; SIVD	↑ clasmatodendrocytes

Legend (in alphabetical order): CADASIL: cerebral autosomal dominant arteriopathy with subcortical infarcts and leukoencephalopathy; SIVD: subcortical ischemic vascular disease; VCI: vascular cognitive impairment; ↑: increase/enhancement; ↓: decrease/reduction; =: no significant change/modification.

**Table 2:** Research agenda and future avenues of translational research in VCI research.

Technique	Potential Applications and Expected Findings
Serum	Screening and advanced protocols that correlate with cognitive performance and progression
	Search for genetic susceptibility for mood disorders associated with vascular pathology
CSF	Screening for early identification of post-stroke cognitive impairment and other VCI subtypes
	Markers of blood–brain barrier involvement or neuroinflammation specifically related to VCI
Neuro- imaging	Implementation and diffusion of advanced structural and functional neuroimaging technique
	Neurosonological markers of early hemodynamic changes in VCI or vascular depression
TMS	Detailed analysis of neurotransmission pathways and other “pharmaco-TMS” studies in VCI
	Advanced brain stimulation protocols to screen and follow mild VCI at risk for dementia
All/Mix	Proposal and application of a multidimensional diagnostic panel to screen population at risk
	Design and optimization of customized cognitive rehabilitation strategies or drug trials

## Abbreviations

AAT	alpha1-antitrypsin
AD	Alzheimer’s disease
ADMA	asymmetric dimethylarginine
APO	apolipoprotein
ASL	arterial spin labelling
Aβ	amyloid-beta
Bax	BCL2 associated X protein
BBB	blood–brain barrier
Bcl-2	B-cell lymphoma 2
BDNF	brain-derived neurotrophic factor
CAA	cerebral amyloid angiopathy
CADASIL	cerebral autosomal dominant arteriopathy with subcortical infarcts and leukoencephalopathy
CBF	cerebral blood flow
CNS	central nervous system
CSF	cerebrospinal fluid
DMA	dimethylarginine
FDG	18-fluorodeoxyglucose
GABA	gamma-amino-butyric acid
G-CSF	granulocyte-colony stimulating factor
H-FABP	heart-type fatty acid binding protein
ICF	intracortical facilitation
IFN-γ	interferon-gamma
IL	interleukin
LBD	Lewy body dementia
L-DOPA	levo-3,4-dihydroxyphenylalanine
MCI	mild cognitive impairment
MIP-1β	macrophage inflammatory protein 1beta
MMP	matrix metalloproteinases
MMSE	Mini Mental State Examination
MRI	magnetic resonance imaging
MRS	magnetic resonance spectroscopy
NF-L	neurofilament-light protein
NO	nitric oxide
PAI-1	plasminogen activator inhibitor 1
PAS	paired associative stimulation
PET	positron emission tomography
PIB	Pittsburgh compound B
P-tau	phosphorilated tau
rMT	resting motor threshold
rTMS	repetitive transcranial magnetic stimulation
SAI	short-latency afferent inhibition
SIVD	subcortical ischemic vascular disease
TCD	transcranial Doppler ultrasound
TIMP-1	tissue inhibitors of metalloproteinases 1
TMS	transcranial magnetic stimulation
TNF-α	tumor necrosis factor alpha
T-tau	total tau
VaD	vascular dementia
VCI	vascular cognitive impairment
VGLUT1	vesicular glutamate transporter 1
WMLs	white matter lesions
